# A Model for Material Metrics in Thermoelectric Thomson Coolers

**DOI:** 10.3390/e25111540

**Published:** 2023-11-14

**Authors:** Mona Zebarjadi, Omid Akbari

**Affiliations:** 1 Department of Electrical and Computer Engineering, University of Virginia, Charlottesville, VA 22904, USA; 2 Department of Materials Science and Engineering, University of Virginia, Charlottesville, VA 22904, USA; 3 Department of Chemical Engineering, University of Virginia, Charlottesville, VA 22904, USA

**Keywords:** Thomson, thermoelectric, figure of merit, analytical model

## Abstract

Thomson heat absorption corresponding to changes in the Seebeck coefficient with respect to temperature enables the design of thermoelectric coolers wherein Thomson cooling is the dominant term, i.e., the Thomson coolers. Thomson coolers extend the working range of Peltier coolers to larger temperature differences and higher electrical currents. The Thomson coefficient is small in most materials. Recently, large Thomson coefficient values have been measured attributed to thermally induced phase change during magnetic and structural phase transitions. The large Thomson coefficient observed can result in the design of highly efficient Thomson coolers. This work analyzes the performance of Thomson coolers analytically and sets the metrics for evaluating the performance of materials as their constituent components. The maximum heat flux when the Thomson coefficient is constant is obtained and the performance is compared to Peltier coolers. Three dimensionless parameters are introduced which determine the performance of the Thomson coolers and can be used to analyze the coefficient of performance, the maximum heat flux, and the maximum temperature difference of a Thomson cooler.

## 1. Introduction

Thermoelectric modules can convert heat to electricity with applications in waste heat recovery [1]. They are also used in heat management techniques such as refrigeration [2,3], active cooling [4,5,6], and thermal switching [7]. A thermoelectric module is made from p-legs and n-legs electrically in series and thermally in parallel. Assuming a temperature independent model for material parameters, including thermal conductivity (κ), electrical conductivity (σ), and Seebeck coefficient (S), a thermoelectric figure of merit, $z=\frac{\sigma S^{2}}{\kappa }$, is defined. It is shown that the efficiency of the thermoelectric power generators and the coefficient of performance of thermoelectric refrigerators are increasing functions of the p-leg and n-leg material figure of merit [8]. While the temperature-independent assumption is only correct under small temperature differences, the simplified, elegant equations for the efficiency allowed the thermoelectric community to systematically scan various materials and estimate their potential for thermoelectric applications based on their z parameter.

There have been many studies identifying the shortcomings in this simplified theory. Finite element modeling is used to include temperature dependence of the material parameters and evaluate modified efficiencies. Functionally graded materials with similar average zT are shown to outperform the temperature-independent materials when used in thermoelectric modules [9,10]. Segmented thermoelectric legs are proposed to improve the efficiency of the thermoelectric modules [11,12,13,14].

Temperature-dependent properties can lead to the design of interesting devices, especially when these dependencies are relatively strong. For instance, one can design a thermal diode using a strong dependence of thermal conductivity on temperature [15]. At a temperature-induced metal–insulator transition when electrical conductivity changes sharply by orders of magnitude, electrical switches can be designed with modern applications in neuromorphic computing [16]. Here, we study the case when the Seebeck coefficient sharply changes with temperature resulting in non-negligible Thomson heating and cooling. Besides the device design, the effect of Thomson cooling is studied in low dimensional silicon nanowires at low temperatures [17], and in the amorphization and temperature rise of phase-change memory cells [18,19], among others.

The Seebeck and Peltier effects are related via the Onsager–Kelvin relation, an example of the Onsager reciprocal relation routed in irreversible thermodynamics, which was demonstrated by W. Thomson (Lord Kelvin), who also showed the presence of a third thermoelectric effect, known as the Thomson effect [20]. In the Thomson effect, heat is absorbed or produced when current flows in a material with a temperature gradient. The heat density (Q) in units of $\frac{W}{m^{3}}$ is proportional to both the electric current (j) and the temperature gradient (∇T) and is expressed as:

(1) Q=−τ j·∇T

The proportionality constant, τ known as the Thomson coefficient, is related by thermodynamics to the Seebeck coefficient as:

(2) $$\tau =T\frac{dS}{dT}$$

This work is motivated by recent observations of sharp changes in the Seebeck coefficient with respect to temperature at phase transition temperature. Modak et al. observed a large Thomson coefficient, τ~1 mV/K, in the FeRh-based alloy system which is associated with the antiferromagnetic–ferromagnetic phase transition [21]. Akhanda et al. reported a Thomson coefficient of τ~100 μV/K at the $T_{d}$ to $1T^{\prime }$ phase transition in MoTe_2_ [22]. Sharp changes in the Seebeck coefficient in Lu_2_Ir_3_Si_5_ at low temperatures were observed which were attributed to a charge–density–wave (CDW) transition [23]. A reconfigurable single material Peltier device is proposed which relies on ferrimagnetic–antiferromagnetic phase transition in Mn_1_._96_Cr_0_._04_Sb where large changes in the Seebeck coefficient at the transition point, are used to design the device [24]. Finally, the effect of Thomson heating and cooling is studied in phase change materials used for memory applications, such as Ge_2_Sb_2_Te_5_ (GST). In this case, the Seebeck coefficient does not change sharply. Instead, it changes almost linearly with temperature. Even though, the Thomson coefficient of the FCC GST films is estimated to be τ~−100 μV/K on the average around the phase transition temperature [25].

In the constant parameter model, the Thomson term is zero. However, if we include this nonlinear contribution to the thermoelectric transport via the inclusion of the temperature-dependent Seebeck coefficient, we observe an extra Joule–Thomson current in the heat diffusion equation resulting in the modified efficiency of thermoelectric modules. Traditionally, thermoelectric coolers are also called Peltier coolers to emphasize the Peltier cooling mechanism. In analogy, and to distinguish between Peltier coolers with negligible Thomson components, we follow the notation used by many and refer to these thermoelectric devices wherein Thomson cooling is the dominant cooling effect as Thomson coolers. We observe distinct behavior in this device compared to a Peltier cooler.

Snyder et al. analyzed self-compatible Thomson coolers where the Thomson term, not the Joule term, predominantly influences the Fourier heat divergence [26]. The self-compatibility in their work implies an exponentially changing Seebeck coefficient with respect to temperature. To maintain the same figure of merit along the device, the product of electrical resistivity and thermal conductivity should be modified accordingly. Under these conditions, the heat conduction equation was solved, and it was shown that self-compatible coolers can outperform the Peltier coolers in terms of achieving a larger maximum temperature difference [26]. More recently, Chiba and coworkers analyzed the temperature profile of a single-element Thomson cooler, wherein the average electrical and thermal conductivity and the Thomson coefficient of the sample were assumed to be constant. They showed that the single-layer system can act as a temperature modulator [27]. Huang et al. studied the temperature distribution of a thermoelectric cooler under the influence of the Thomson effect, Joule heating, Fourier’s heat conduction, and radiation [28]. Sun et al. studied the effect of the Thomson term numerically on the performance of micro-thermoelectric coolers [29]. They showed that a positive Thomson coefficient can improve the cooling capacity and a higher current and thickness, a smaller thickness to the cross-section area, and a larger cooling load corresponding to a greater impact of the Thomson effect. Gong et al. developed a one-dimensional thermodynamic model to evaluate the device-level performance of a thermoelectric cooler (TEC) with the Thomson effect. Similarly, they observed a greater influence of the Thomson effect on cooling capacity with the increasing current [30].

Despite these efforts, we still do not have an equivalent figure of merit defined for Thomson coolers and power generators. The purpose of this work is to evaluate the performance of Thomson coolers and define an equivalent figure of merit.

## 2. Analysis

### 2.1. Thermoelectric Modules

We start by reminding the readers about the performance of a thermoelectric Peltier cooler. While this analysis can be found in many textbooks, here, for the sake of complicity, we have included the detailed analysis in the Supplementary Materials.

**Figure 1:**
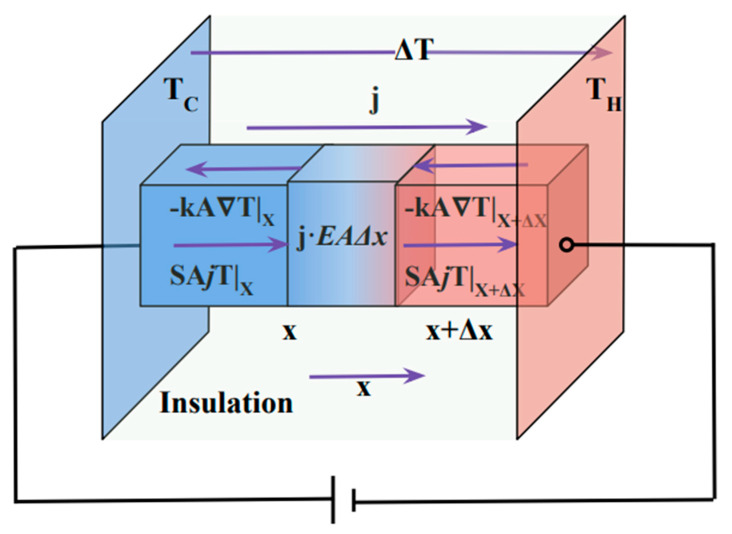
Schematic diagram of heat balance in a Thomson/Peltier cooler.

A Peltier module is made from alternating p and n legs. However, writing the equations for two legs or one (ignoring thermal and electrical resistances and losses in connecting metals) results in similar equations. Hence, to simplify here, we only include one p-type thermoelectric leg. We assume perfect insulation from the side walls and hence a one-dimensional heat and electricity transport along the length of the device (*x*-axis). Figure 1 shows the schematic of the heat flux within a p-type leg of a Peltier/Thomson cooler.

Heat flux ($q\left(x\right)$ in units of $\frac{W}{m^{2}}$) under temperature gradient, $\frac{dT}{dx}$, and electric current, j, and for a 1D transport can be written as:

(3) $$q\left(x\right)=-\kappa \frac{dT}{dx}+jS(x)T(x)\;$$

Assuming temperature-independent parameters, S and κ are constants. By writing the heat balance on a segment of a thermoelectric leg (see Supplementary Materials and Figure 1), we can obtain the heat conduction equation:

(4) $$\kappa \frac{d^{2}T}{dx^{2}}+\rho j^{2}=0$$

The first term represents the heat conduction, and the second term represents Joule heating with ρ representing the resistivity. This equation can be solved given the boundary conditions that depend on the device’s operational mode. For instance, if the purpose is to maximize the temperature difference of a thermoelectric cooler, then one must set the boundary conditions as heat flux at the cold side (x=0) to be zero: $q\left(x=0\right)=0$ and the temperature at the cold side to be fixed $T\left(x=0\right)=T_{c}$ to find the temperature profile (solution of Equation (4)). Upon calculation of the temperature difference and optimization with respect to the current density, it can be shown that the maximum temperature difference is proportional to the thermoelectric figure of merit:

(5) $$\Delta T_{mx}=\frac{zT_{C}^{2}}{2}$$

This is perhaps the fastest way to find the figure of merit form in a thermoelectric cooler. This same metric shows up in the calculation of the coefficient of performance (COP) of coolers and the efficiency of power generators. We refer the reader to the Supplementary Materials and many available textbooks [31,32] for more details.

### 2.2. Thomson Modules

To be able to analyze Thomson modules, let us start by assuming κ and ρ are constant (temperature independent) while the Seebeck coefficient changes with temperature.

The heat flux (Equation (3)) is unchanged, but the heat conduction equation (Equation (4)) is now modified to:

(6) $$\kappa \frac{d^{2}T}{dx^{2}}+\rho j^{2}-j\frac{dS}{dT}T\frac{dT}{dx}=0$$

Here, the first two terms are the same as before but now we have a new third term, the so-called Joule–Thomson term [20]. To progress, we go with the simplest assumption for the temperature dependence of the Seebeck coefficient and we take the $\tau =\frac{dS}{dT}T$ to be constant. We note that this implies that the Seebeck coefficient has a logarithmic form with respect to temperature:

(7) $$\kappa \frac{d^{2}T}{dx^{2}}+\rho j^{2}-j\tau \frac{dT}{dx}=0$$

To proceed, we assume the Thomson coefficient is positive for this p-type sample. Taking the *x*-axis as shown in Figure 1 in the direction of the current, the third term in Equation (7) is negative only if the Thomson coefficient is positive which implies the Thomson term serves as heat absorption to lower the Joule heating. For a negative τ, the third term works along with the Joule heating term and lowers the efficiency of the cooler by generating extra heat, hence, is not useful.

The general solutions of Equation (7) can be written as:

(8) $$T\left(x\right)=\frac{\rho j}{\tau }x+C_{1}e^{j\tau x/\kappa }+C_{2}$$

where the temperature increase is exponentially along the *x*-axis and $C_{1}$ and $C_{2}$ are constant parameters and are dependent on the boundary conditions.

#### 2.2.1. Maximum Temperature Difference

To obtain the figure of merit for Thomson coolers, let us maximize the temperature difference by setting the heat flux at the cold side to zero. Upon some algebraic steps which are detailed in Supplementary Materials, we obtain:

$$\Delta T=\frac{\rho j}{\tau }L+(-\kappa \frac{\rho }{\tau^{2}}+\frac{S_{C}T_{C}}{\tau })\left(e^{\frac{j\tau L}{\kappa }}-1\right)$$

Here, we note that we have several possibilities to define the figure of merit, including $\frac{\tau S}{\rho \kappa }$ and $\frac{\tau^{2}}{\rho \kappa }$. However, we choose to keep the figure of merit to be that of the thermoelectric figure of merit to be able to compare it to a normal thermoelectric module. By doing so, we obtain:

(9) $$\Delta T=\frac{1}{z\alpha^{2}}[\gamma +\left(-1+\alpha_{C}\;z_{C}T_{C}\right)\left(e^{\gamma }-1\right)]$$

Here, $z_{C}$ refers to the thermoelectric figure of merit on the cold side. We note that since the Seebeck coefficient changes along the device while the other properties are constant, the figure of merit, z, is not constant and follows the Seebeck changes. A secondary parameter ($\alpha =\frac{\tau }{S}$) is present in Equation (9), which is different from the thermoelectric case. This dimensionless parameter can be combined with the thermoelectric figure of merit to recover other choices of the Thomson figure of merit seen above, i.e., $z\alpha =\frac{\tau S}{\rho \kappa }$ and $z\alpha^{2}=\frac{\tau^{2}}{\rho \kappa }$. Finally, a third dimensionless parameter γ is the dimensionless current: $\gamma =\frac{j\tau L}{\kappa }$. Next, we need to optimize the temperature difference with respect to γ to obtain:

(10) $$\Delta T_{mx}=-\frac{1}{z\alpha^{2}}[\ln\left(1-\alpha_{C}\;z_{C}T_{C}\right)+\alpha_{C}\;z_{C}T_{C}]$$

If we take the limit of α→0, we recover $\Delta T_{mx}=\frac{zT_{c}^{2}}{2}$ which is the thermoelectric limit.

The form obtained here for the maximum temperature difference only works for small values of zαT. It is divergent when zαT=1 and is complex for larger values which are not physical. When zαT≥1, Equation (9) is an increasing function of current and does not have a maximum. This does not happen in a real device as the Seebeck coefficient will not increase with temperature logarithmically and over large temperature differences. Despite that, this analysis helped us identifying the two important material parameters for Thomson coolers. The thermoelectric figure of merit and the ratio of the Thomson coefficient to the Seebeck coefficient (α) are the main parameters determining the functionality of a Thomson cooler. The third dimensionless parameter, γ, adjusts the optimum electrical current density when there is one. With these dimensionless parameters in mind, let us now optimize the heat flux and the COP.

#### 2.2.2. Maximum Heat Flux

In this section, we study the optimum heat flux on the cold side. We start from the general solutions of the heat conduction equation as expressed in Equation (8). We first set the boundary conditions to fixed temperatures on both sides. We note that since we are only dealing with temperature gradient, the value of $C_{2}$ is not needed:

(11) $$T\left(x\right)=\frac{\rho j}{\tau }x+(\frac{\Delta T-\frac{\rho j}{\tau }L}{e^{\gamma }-1})e^{j\tau x/\kappa }+C_{2}$$

The heat flux at the cold side (x=0) is:

(12) $$q_{c}=-\kappa \nabla T{\left.\right|}_{x=0}+jS_{c}T_{c}=-\kappa \frac{\rho j}{\tau }-j\tau \frac{\Delta T-\frac{\rho j}{\tau }L}{e^{\frac{j\tau L}{\kappa }}-1}+jS_{c}T_{c}$$

Next, we maximize q with respect to the current to obtain the maximum heat flux on the cold side. This is achieved by expanding Equation (12) in the polynomial series with respect to j and keeping only up to the second-order term. In the Supplementary Materials, we show that the third-order term is much smaller than the second-order term in most practical cases. In extreme cases, this assumption may be violated. The following are the criteria for the analysis to be correct: $\alpha^{2}z\Delta T\ll 6$ and γ≪6. We note that the first criterion is satisfied in most materials and conditions since in most cases zT<2, ΔT≪T, and α<1. The second criterion, assuming the following orders of magnitude: $\tau \sim 10^{-4}\frac{V}{K}$, $\kappa \sim 1\frac{W}{mK}$, $L\sim 10^{-3}m$, and $j\sim 10^{5}\frac{A}{m^{2}}$, is also valid since $\gamma \sim 10^{-3}\ll 1$. However, it can break when the Thomson coefficient is large, for long device lengths, or large applied currents. After expansion, we can optimize the flux with respect to current, j. Since we now have a second-order polynomial, it is feasible to set its derivative with respect to j to zero and solve for the optimum heat flux. Upon doing so, we obtain:

(13) $$q_{opt}=\frac{\kappa \Delta T}{L}[\frac{-z\alpha^{2}\Delta T+12\alpha_{c}z_{C}T_{C}-24+12z_{C}T_{C}^{2}/\Delta T}{4\left(z\alpha^{2}\Delta T+6\;\right)}]$$

If we take the limit of α→0, we recover the optimum heat flux of a thermoelectric cooler:

(14) $$q_{opt}^{TE}=\frac{\kappa \Delta T}{L}[\frac{z_{C}T_{C}^{2}}{2\Delta T}-1]$$

**Figure 2:**
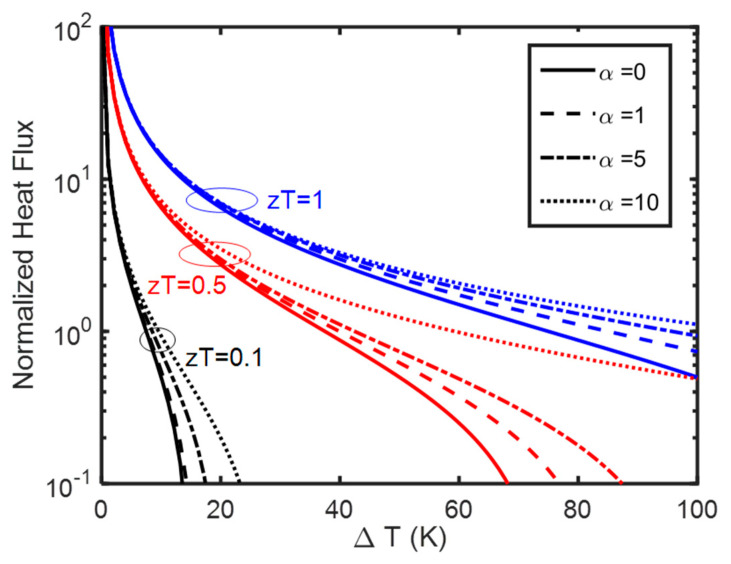
Normalized optimum heat flux at the cold side ($q_{opt}/\frac{\kappa \Delta T}{L})$ plotted for $T_{c}=300\;K$. Solid lines are for Peltier coolers (Equation (13), α=0). zT values are shown for each series of curves with the same color. Dashed lines are Thomson coolers for α=1, dotted dash for α=5 and dotted lines for α=10. In each series, we keep the zT value like the Peltier coolers and we increase the α ratio.

Let us study when the Thomson cooler can pump noticeably more heat flux compared to normal thermoelectric Peltier coolers. To do so, we plot the normalized heat flux ($q_{opt}/\frac{\kappa \Delta T}{L})$ at the cold side as calculated in Equation (13) for Thomson coolers and Equation (14) for Peltier coolers as a function of ΔT. When this heat flux reaches negative values, heat goes in the natural heat flux direction and from hot to cold with −1 recovering the Fourier’s law. The heat pump works properly when the heat flux values are positive indicating the heat is pumped from cold to hot. Figure 2 shows the results when $T_{c}$ is kept at 300 K and for different zT and α values. As we use the logarithmic axis, the negative values are not shown but happen at large temperature differences. At a given zT, as we increase α, the heat flux increases as expected. We see a larger improvement when temperature differences are larger. Percentage-wise, the improvement (the ratio of the heat flux in the Thomson cooler divided by the Peltier cooler at a given ΔT) is larger when zT is smaller and the difference decreases at larger zT values, but this might be artificial as the results for larger zT and larger α values may violate the conditions under which Equation (13) was obtained. Numerical modeling is needed to confirm the validity of the data at higher zT and α values. Hence, we optimize Equation (12) numerically. The original form has too many parameters, and we need to minimize them. Rewriting Equation (12) in dimensionless form we obtain:

(15) $$\frac{q\left(x=0\right)L}{\kappa \Delta T}=-\Upsilon \frac{1-\frac{\Upsilon }{z\alpha^{2}\Delta T}}{e^{\Upsilon }-1}+\frac{\Upsilon T_{c}}{\alpha_{c}\Delta T}\;\;\left(1-\frac{1}{\alpha_{c}z_{c}T_{c}}\right)$$

**Figure 3:**
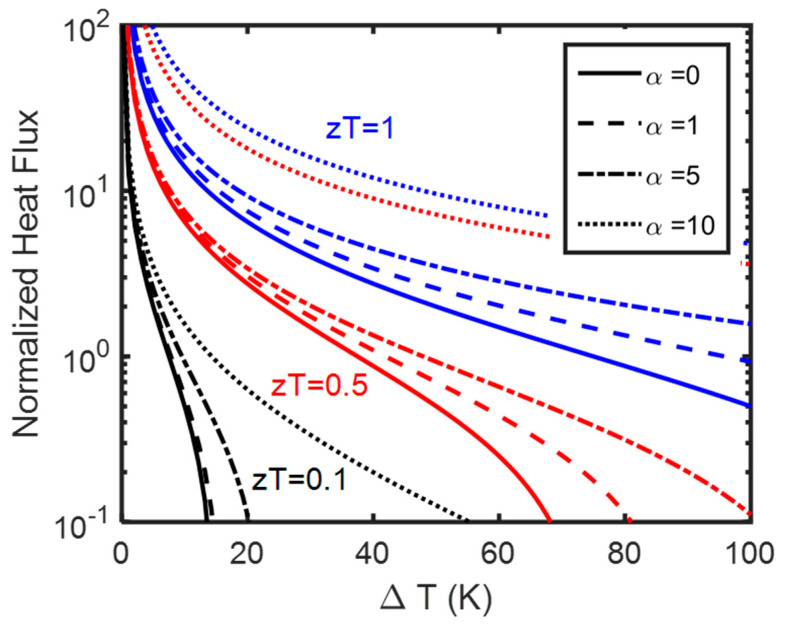
Numerical results of heat flux optimization. Normalized optimum heat flux at cold side ($q_{opt}/\frac{\kappa \Delta T}{L})$ plotted for $T_{c}=300\;K$. Solid lines are for Peltier coolers (Equation (13), α=0). zT values are shown for each series of curves with the same color. Dashed lines are Thomson coolers for α=1, dotted dash for α=5 and dotted lines for α=10. In each series, we keep the zT value like the Peltier coolers and we increase the α ratio.

As noted before, the Seebeck coefficient and therefore α and z are position-dependent. However, $z\alpha^{2}$ is position-independent and we can drop the sub-index, C, referring to the cold side. Next, we optimize this function (Equation (15)) with respect to the current. We note the parameter Υ, does not affect the optimum heat flux significantly but rather changes the value of the optimum current. The larger $\Upsilon /j=\frac{\tau L}{\kappa }$ values correspond to smaller optimum current values, implying that coolers made with larger Thomson coefficients and smaller thermal conductivity values can operate under smaller optimum electrical currents. Let us now recreate Figure 2 numerically. The results using the same format are plotted in Figure 3. At small zT and α values, numerical results and analytical results match. However, at large zT values and large α values, numerically, we observe a much larger improvement in the optimum heat flux compared to Peltier coolers (e.g., see Figure 3 in comparison with Figure 2 when zT = 1). Numerically we can confirm that given all parameters being the same, the performance is always better when the Thomson coefficient is larger. However, the improvement in small zT values is still larger percentagewise which is consistent with the analytical results of Figure 2.

#### 2.2.3. Maximum Coefficient of Performance, COP

Finally, we attempt to maximize the COP of Thomson coolers and study the difference between Peltier and Thomson coolers. The COP is defined as the ratio of the heat flux on the cold side, $q_{c},$ divided by the electric work performed, $P_{E},$ where

(16) $$P_{E}=q_{H}-q_{c}$$

(17) $$COP=\frac{q_{c}}{P_{E}}=\frac{\left[-j\frac{\tau \Delta T-\rho Lj}{{\mathrm{e}}^{j\frac{\tau }{\kappa }L}-1}+j\;S_{C}T_{C}\left(1-\frac{1}{z_{c}T_{c}\alpha_{c}}\right)\right]}{\left[-j\tau \frac{\Delta T-\frac{\rho j}{\tau }L}{e^{\frac{j\tau L}{\kappa }}-1}(e^{\frac{j\tau L}{\kappa }}-1)+j(S_{H}T_{H}-S_{c}T_{c})\right]}=\frac{\left[-\frac{\Delta T-\frac{\gamma }{z\alpha^{2}}}{{\mathrm{e}}^{\gamma }-1}+\frac{T_{C}}{\alpha_{C}}\left(1-\frac{1}{z_{c}T_{c}\alpha_{c}}\right)\right]}{\left[-\Delta T+\frac{\gamma }{z\alpha^{2}}+(\frac{T_{H}}{\alpha_{H}}-\frac{T_{c}}{\alpha_{c}})\right]}$$

Similar to Equation (15), we have three independent dimensionless parameters γ, zT, and α. However, there is a new parameter $\alpha_{H}$ referring to the Thomson ration on the hot side. This parameter is not independent of other defined parameters and can be expressed in terms of $\alpha_{c}$. We assumed TdS/dT=τ which indicated that $S=\tau lnT+S_{0}$. Using this relation, we obtain:

(18) $$\frac{1}{\alpha_{H}}-\frac{1}{\alpha_{c}}=\frac{S_{H}}{\tau }-\frac{S_{c}}{\tau }=lnT_{H}-lnT_{c}$$

**Figure 4:**
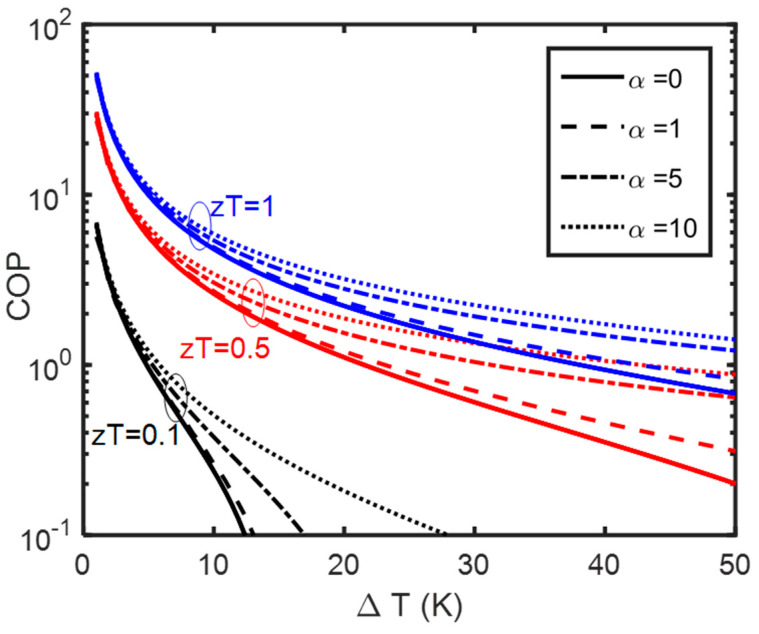
Numerical results of COP optimization plotted for $T_{c}=300\;K$. Solid lines are for Peltier coolers. ZT values are shown for each series of curves and with the same color. Dashed lines are Thomson coolers. In each series, we kept the zT value the same as that of the Peltier coolers and we increased the α ratio.

Therefore, we can use the values of the dimensionless parameters on the cold side to obtain the COP. We then optimize the COP with respect to the current numerically. Figure 4 shows the resulting optimum COP values as a function of temperature difference and using the same style as Figure 2 and Figure 3. Here, we see a similar trend of the COP increasing both with zT and α and improving more percentage-wise at smaller values.

## 3. Thomson Materials

Finally, we study the performance of some of the reported materials as Thomson cooler constituent materials. There are only a handful of materials that are studied specifically for their Thomson coefficient. One of the most recent examples is the Fe-Rh alloy [21], reported last year to have a small Seebeck coefficient or −11 μV/K but a very large Thomson coefficient of −906 μV/K. The Thomson coefficient has a bell shape with respect to temperature and is only large in a narrow temperature window of about 20 K. The corresponding Thomson ratio (α) is large and is around 87. However, the thermoelectric zT is very small and on the order of $10^{-3}$. Defining the Thomson figure of merit as zαT, the value is 0.2 and it is not large enough to produce a substantial performance. Likewise, Mn_1.96_Cr_0.04_Sb has a very small Seebeck value of 7.5 μV/K, which is rapidly changing at its magnetic phase transition, corresponding to a Thomson coefficient of ~180 μV/K within a 5 K window [24]. MoTe_2_ is reported to have an α value of 5 at its 1T-Td phase transition [22]. Sr_6_Co_5_O_15_ is reported to have a Seebeck coefficient of ~125 μV/K at 875 K which is increasing in the range of 700 K to 1000 K with an estimated Thomson coefficient of 98 μV/K [33]. Bi_88_Sb_12_ is reported to have a Thomson coefficient of 45 μV/K which increases to 98 μV/K when the magnetic field is applied [34]. In all these cases, the material is not primarily optimized for thermoelectric applications and thermoelectric zT is small. The Thomson zT is larger but not enough to observe a significant advantage compared to traditional thermoelectric materials.

The case of CuSe_2_ is an exceptionally interesting one that was reported in 2019 for the giant thermoelectric zT as a result of a colossal Seebeck coefficient [35]. The Seebeck coefficient values exceed ±2  mV/K in a narrow temperature range, 340 K < T < 400 K, where a structural phase transition takes place. The thermoelectric zT is a delta function with a pick value larger than 400! The Thomson coefficient in this case is even larger than the Seebeck coefficient itself, and hence the overall Thomson figure of merit is exceptionally large. The Seebeck coefficient starts from small positive values at below 320 K and increases in magnitude to negative values up to 340–350 K. The window in which the Seebeck is negative, and the increase in its magnitude is only about 10 K which is the useful window for the Thomson application. The fact that the Thomson figure of merit is large in a narrow window suggests that Thomson coolers designed based on these materials should work better in transient regimes and with short pulses, as a result of which they can absorb a large amount of heat in a short period. The heat should be pumped fast and precisely to prevent temperature increases beyond the phase transition window after which the figure of merit is low, and the cooler is inefficient.

## 4. Conclusions

In this work, we analyzed the performance of Thomson coolers analytically when the Thomson coefficient is temperature-independent. This corresponds to when the Seebeck coefficient is changing logarithmically with temperature. There are several different ways to define the Thomson figure of merit. The dimensionless parameters determining the performance are in the format of $\frac{S^{2}T}{\rho \kappa }$, $\frac{\tau ST}{\rho \kappa }$, and $\frac{\tau^{2}T}{\rho \kappa }$ which are related to the thermoelectric figure of merit and α=τ/S parameters. The coefficient of performance and the maximum heat flux are increasing functions of both zT and α. A third parameter $\Upsilon /j=\frac{\tau L}{\kappa }$, determines the optimum current density. Larger Υ values are desired as they correspond to smaller optimum currents, lowering the overall Joule heating. The Thomson coolers can extend the performance of the Peltier coolers to larger temperature differences. A positive Thomson coefficient is desired for p-type semiconductors and a negative one for n-type semiconductors. When studying the percentage improvement, i.e., the ratio of the Thomson maximum heat flux to the Peltier maximum heat flux, and when all parameters are the same, the percentage improvement is larger at smaller z values. However, when studying the absolute values, we note that the Thomson coolers are advantageous at larger z and α values.

## Data Availability

The data presented in this study are available on request from the corresponding author.

## Supplementary Materials

The following supporting information can be downloaded at: https://www.mdpi.com/article/10.3390/e25111540/s1.
