# Supplementary material for: A Model for Material Metrics in Thermoelectric Thomson Coolers
Source: Entropy (Basel). 2023 Nov 14;25(11):1540. doi: 10.3390/e25111540 (PMC10670593; doi:10.3390/e25111540)
Supplement: Supplementary file 1 [file entropy-25-01540-s001.zip › entropy-2691718-supplementary.pdf]

Supplementary materials

I. Symbols:

S : Seebeck coefficient
τ: Thomson coefficient
ρ: electric resistivity
κ: thermal conductivity
α: Ratio of Thomson to Seebeck
A: Area
l: Length
$T_{H/C}$: Temperature of hot/cold side
ΔT: Temperature difference

I: Electrical Current
j: Electrical Current density
$Q_{H/C}$: Thermal rate at hot/cold side
$q_{H/C}$: Thermal flux at hot/cold side
PE: Work (electric power)
COP: Coefficient of Performance
Z: Figure of merit
ZT: dimensionless figure of merit
PF: TE power factor (W/m^2K)
PFT: TE power factor (W/mK)

a) Thermoelectric Refrigerator:

Let's start with the more familiar TE refrigerator. Heat is pumped from the cold side to the hot side by applying current. Insulation is used between the p-n legs to enforce the heat transfer only through the thermoelectric legs. Therefore, boundary conditions are zero heat flux on the sides and fixed temperature at hot and cold ends.

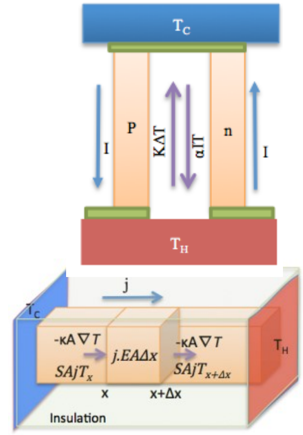

FIG. S1. Schematic of a thermoelectric refrigerator is shown on top and detailed energy balance for the p leg is shown in the lower graph.

We can write the heat conduction equation for one leg (p-leg) by writing the energy balance for a slab as shown schematically in figure S1. Consider the electrons and phonons as a single system so that we do not need to deal with the internal energy exchanges between electrons and phonons. Total thermal current at each position x (including electron and phonon contributions could be written as:

$$Q_x = -\kappa A \nabla T_x + SIT_x \qquad \text{S 1}$$

Therefore the heat balance equation for the schematically shown slab could be written as:

$$-\kappa A \nabla T|_x + \kappa A \nabla T|_{x+\Delta x} + SAjT_x - SAjT_{x+\Delta x} + j.EA\Delta x = 0 \qquad \text{S 2}$$

Noting that under temperature gradient and applied electric field E, the coupled current equation for electron-phonon system with electrical (j) and total heat flux (electrons and phonons, q) could be written as:

$$j = E/\rho - S/\rho\, \nabla T \qquad \text{S 3}$$

$q = -\kappa \nabla T + STj$ S 4

If we write E from S 3 in terms of j and ∇T and substitute in S 2 then divide by $A\Delta x$, we find:

$$\frac{\mathrm{d}^2 T}{dx^2} + \frac{\rho j^2}{\kappa} = 0 \quad \text{S 5}$$

With boundary conditions of $T = T_C$ $(x = 0)$ and $q(x = 0) = 0$

We find

$$T(x) = -\frac{\rho j^2}{2\kappa} x^2 + b\, x + T_C \quad \text{S 6}$$

$$q_C|_p = q_{x=0} = -\kappa b + SjT_C = 0 \rightarrow b = SjT_C/\kappa \quad \text{S 7}$$

$$T(x) = -\frac{\rho j^2}{2\kappa} x^2 + \frac{SjT_C}{\kappa} x + T_C \quad \text{S 8}$$

$$T_H - T_c = \Delta T = -\frac{\rho j^2}{2\kappa} L^2 + \frac{SjT_C}{\kappa} L \quad \text{S 9}$$

$$\frac{\mathrm{d}T}{dj} = -\frac{\rho j}{\kappa} L^2 + \frac{ST_C}{\kappa} L = 0 \rightarrow j_{opt} = \frac{ST_C}{\rho L} \rightarrow \Delta T_{mx} = \frac{zT_C^2}{2} \quad \text{S 10}$$

b) Thomson cooler

It is said that the Thomson coolers work by only one leg and do not require n-p geometry. The purpose of this analysis is to understand how they work and define an equivalent figure of merit if possible. Here to simplify, we use a one-leg geometry and keep it p-type. The analysis is the same as before but now S is temperature dependent.

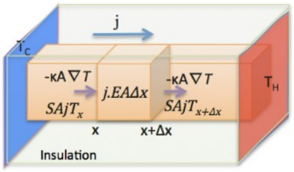

FIG S2. P-type Thomson cooler

Total thermal current at each position x (including electron and phonon contributions could be written as:

$$Q_x = -\kappa A \nabla T_x + SIT_x \quad \text{S 11}$$

Therefore the heat balance equation for the schematically shown slab could be written as:

$$-\kappa A \nabla T|_x + \kappa A \nabla T|_{x+\Delta x} + AjS_x T_x - AjS_{x+\Delta x} T_{x+\Delta x} + j.EA\Delta x = 0 \quad \text{S 12}$$

Noting that under temperature gradient and applied electric field E, the coupled current equation for electron-phonon system with electrical (j) and total heat flux (electrons and phonons, Q') could be written as:

$$j = \frac{E}{\rho} - \frac{S}{\rho} \nabla T \rightarrow E = \rho j + S \nabla T \quad \text{S 13}$$

If we write E from S3 in terms of j and ∇T and substitute in S2 then divide by $A\Delta x$, we find:

$$\kappa\nabla^2 T - j\frac{d}{dx}(ST) + j.E = 0 \qquad \text{S 14}$$

Here, we need to use the average E inside the element. But this means average S and will make it complex. So for the sake of simplicity, let's use S and not average S.

$$\kappa\nabla^2 T - j\frac{d}{dx}(ST) + \rho j^2 + Sj\nabla T = 0 \qquad \text{S 15}$$

$$\kappa\nabla^2 T - j\frac{dS}{dx}T + \rho j^2 = 0 \rightarrow \quad \kappa\nabla^2 T - j\frac{dS}{dT}T\frac{dT}{dx} + \rho j^2 = 0 \qquad \text{S 16}$$

We need to assume something for S to enable continuing.

<u>**Assuming:**</u> $\tau = T\frac{dS}{dT} = constant$ and positive

$$\kappa\nabla^2 T - j\tau\,\nabla T + \rho j^2 = 0 \quad \text{or } T'' - j\,\tau/\kappa\, T' + \rho j^2/\kappa = 0 \qquad \text{S 17}$$

The general solutions are

$$T(x) = \frac{\rho j}{\tau}x + C_1 e^{j\tau x/\kappa} + C_2 \qquad \text{S 18}$$

$$\nabla T(x) = \frac{\rho j}{\tau} + \frac{j\tau}{\kappa}e^{j\tau x/\kappa}C_1 \qquad \text{S 19}$$

1.1-With boundary conditions of $T = T_C\ (x=0)$ and $q(x=0) = 0$

$$T_c = C_1 + C_2 \qquad \text{S 20}$$

$$q_C = q_{x=0} = -\kappa\nabla T_{x=0} + jS_C T_C = -\kappa\frac{\rho j}{\tau} - j\tau C_1 + jS_C T_C = 0 \rightarrow -\kappa\frac{\rho}{\tau^2} + \frac{S_C T_C}{\tau} = C_1 \qquad \text{S 21}$$

$$\Delta T = \frac{\rho j}{\tau}L + C_1\left(e^{\frac{j\tau L}{\kappa}} - 1\right) = \frac{\rho j}{\tau}L + \left(-\kappa\frac{\rho}{\tau^2} + \frac{S_C T_C}{\tau}\right)\left(e^{\frac{j\tau L}{\kappa}} - 1\right) \qquad \text{S 22}$$

$$\frac{dT}{dj} = \frac{\rho}{\tau}L + \left(-\kappa\frac{\rho}{\tau^2} + \frac{S_C T_C}{\tau}\right)\left(\frac{\tau L}{\kappa}e^{\frac{j\tau L}{\kappa}}\right) = 0 \rightarrow \frac{1}{\left(1-\frac{\tau S_C T_C}{\rho\kappa}\right)} = e^{\frac{j\tau L}{\kappa}} \rightarrow -\frac{\kappa}{\tau L}\ln\left(1 - \frac{\tau S_C T_C}{\rho\kappa}\right) = j \qquad \text{S 23}$$

$$\Delta T_{mx} = -\frac{\kappa\rho}{\tau^2}\ln\left(1 - \frac{\tau S_C T_C}{\rho\kappa}\right) - \left(\frac{S_C T_C}{\tau}\right) = -\frac{\kappa\rho}{\tau^2}\left[\ln\left(1 - \frac{\tau S_C T_C}{\rho\kappa}\right) + \left(\frac{\tau S_C T_C}{\kappa\rho}\right)\right] \qquad \text{S 24}$$

$$\lim_{\tau\to 0}\Delta T_{mx} = \lim_{\tau\to 0}\left[-\frac{\kappa\rho}{2\tau}\left[\frac{-\frac{S_C T_C}{\rho\kappa}}{\left(1-\frac{\tau S_C T_C}{\rho\kappa}\right)} + \left(\frac{S_C T_C}{\kappa\rho}\right)\right]\right] = \lim_{\tau\to 0}\left[\frac{1}{2}\frac{S_C^2 T_C^2}{\rho\kappa}\left[\frac{1}{\left(1-\frac{\tau S_C T_C}{\rho\kappa}\right)}\right]\right] = \frac{1}{2}\frac{S_C^2 T_C^2}{\rho\kappa} = \frac{1}{2}zT_c^2 \qquad \text{S 25}$$

1.2-With boundary conditions of $T = T_C$ $(x = 0)$ and $T = T_H \; at \; x = L$

$$T(x) = \frac{\rho j}{\tau}x + C_1 e^{j\tau x/\kappa} + C_2 \qquad \text{S 26}$$

$$T(x = 0) = T_C = C_1 + C_2 \qquad \text{S 27}$$

$$T(x = L) = T_H = \frac{\rho j}{\tau}L + C_1 e^{j\tau L/\kappa} + C_2 \qquad \text{S 28}$$

$$\frac{\Delta T - \frac{\rho j}{\tau}L}{e^{\frac{j\tau L}{\kappa}} - 1} = C_1 \qquad \text{S 29}$$

$$\nabla T = \frac{\rho j}{\tau} + \frac{j\tau}{\kappa}\frac{\Delta T - \frac{\rho j}{\tau}L}{e^{\frac{j\tau L}{\kappa}} - 1}e^{j\tau x/\kappa} \qquad \text{S 30}$$

$$q = -\kappa\nabla T + jST = -\kappa\frac{\rho j}{\tau} - j\tau\frac{\Delta T - \frac{\rho j}{\tau}L}{e^{\frac{j\tau L}{\kappa}} - 1}e^{j\tau x/\kappa} + jST \qquad \text{S 31}$$

Details for maximizing the heat flux:

$$q(x = 0) = -j\frac{\tau\,\Delta T - \rho j L}{e^{j\frac{\tau}{\kappa}L} - 1} + j\left(S_c T_c - \frac{\rho\kappa}{\tau}\right) = -\tau j\frac{\Delta T - \frac{\rho L j}{\tau}}{e^{j\frac{\tau}{\kappa}L} - 1} + jS_c T_c\left(1 - \frac{1}{z_c T_c \alpha_c}\right) \qquad \text{S 32}$$

Let us call $R = \rho L$, The following parameter is dimensionless: Rj/ST

$$q(0) = -j\frac{\tau\Delta T - Rj}{e^{j\frac{\tau}{\kappa}L} - 1} + j\,S_C T_C\left(1 - \frac{1}{z_C T_C \alpha_C}\right) = -j\frac{A' - Rj}{e^{jC'} - 1} + j\,B' \qquad \text{S 33}$$

Upon expansion around j:

$$q(0) = -\frac{A'}{C'} + \left(\frac{A'}{2} + B' + \frac{R}{C'}\right)j + \left(-\frac{A'C'}{12} - \frac{R}{2}\right)j^2 + \frac{1}{12}C'Rj^3 + O[j]^4 \qquad \text{S 34}$$

The simplest is to keep only up to the second-order term. To do so we look at the ratio of the third-order term to the second-order term

$$Ratio\ of\ the\ terms = \frac{\frac{1}{12}C'R\mathrm{j}}{\frac{A'C'}{12} + \frac{R}{2}} = \frac{\frac{\tau}{\kappa}L\mathrm{j}}{\frac{\tau^2\Delta T}{\rho\kappa} + 6} = \frac{\frac{\tau}{\kappa}L\mathrm{j}}{(\alpha^2 z\Delta\mathrm{T} + 6)}$$

In most cases $\alpha^2 z\Delta T \ll 6$

And we take zT ~ 1, set $j < 100\frac{A}{cm^2}$ and take the order of magnitude for the other parameters, i.e. $S \sim 10^{-4}\frac{V}{K}$, $\kappa \sim 1\frac{W}{mK}$, and $L \sim 1mm$

Then $ratio \sim \frac{\tau}{6\kappa}L\mathrm{j} < \frac{10^{-4}\frac{V}{K}\,10^{-3}\,m\,10^{6}\frac{A}{m^2}}{6\;1\frac{W}{mK}} < 0.016$

$$q(0) = -j\frac{\tau\Delta T - Rj}{e^{j\frac{\tau}{\kappa}L}-1} + j\, S_C T_C \left(1 - \frac{1}{z_C T_C \alpha_C}\right) = -j\frac{A' - Rj}{e^{jC'}-1} + j\, B' \qquad B' = \left(S_C T_C - \frac{S_C}{z_C \alpha_C}\right)$$

$$B'C' = \frac{\tau}{\kappa} L \left(S_C T_C - \frac{S_C}{z_C \alpha_C}\right) = \left(S_C T_C \frac{\tau}{\kappa} L - R\right)$$

$$A'C' = \Delta T \frac{\tau^2}{\kappa} L$$

$$q_{opt} = \frac{-A'^2 C'^2 + 12A'C'(B'C' - R) + 12(B'C' + R)^2}{4C'^2(A'C' + 6R)} = \frac{-(\tau^2 \Delta T^2) + 12\Delta T \kappa (S_C T_C \frac{\tau}{\kappa} - 2\rho) + 12(S_C T_C)^2}{4(\Delta T \frac{\tau^2}{\kappa} L + 6R)} \qquad \text{S 35}$$

$$q_{opt} = \frac{\kappa \Delta T}{L}\left[\frac{-z\alpha^2 \Delta T + 12\alpha_C z_C T_C - 24 + 12 z_C T_C^2/\Delta T}{4(z\alpha^2 \Delta T + 6\,)}\right] \qquad \text{S 36}$$

If $\alpha \to 0$

$$q_{opt} = \frac{\kappa \Delta T}{L}\left[-1 + z_C T_C^2 / 2\Delta T\right] \qquad \text{S 37}$$

While this works well, I noted that at larger values of zT and Thomson, the results are strange. Perhaps the expansion is not valid anymore and higher-order terms are needed. We can try to make the original heat flux dimensionless and see if it helps

$$q(0) = -j\frac{\tau\Delta T - Rj}{e^{\gamma}-1} + j\, \left(S_C T_C - \frac{S_C}{z_C \alpha_C}\right)$$

Doing the same process as before and maximizing the heat flux after keeping the third-order term results in the following expression which is too long to be useful.

$$\frac{q}{\kappa} = \Big(\frac{-\Delta T^4 \tau^6 + 72\kappa\,\rho\, S^2 T^2 (6\,\kappa\,\rho + ST\tau) + 3\Delta T^3 \tau^4(-9\kappa\rho + 4ST\tau) - 36\,\Delta T\,\kappa\rho(24\kappa^2\rho^2 - 12\kappa\rho ST\tau - 7S^2T^2\tau^2)}{4L(6\kappa\rho + \Delta T\,\tau^2)^3} +$$

$$\frac{6\Delta T^2 \tau^2(-54\kappa^2\rho^2 + 33\kappa\rho ST\tau + 2S^2T^2\tau^2))}{4L(6\kappa\rho + \Delta T\,\tau^2)^3}$$

Hence it is best to solve the heat flux numerically as done in the manuscript.
